# Duration of Immunity to Norovirus Gastroenteritis

**DOI:** 10.3201/eid1908.130472

**Published:** 2013-08

**Authors:** Kirsten Simmons, Manoj Gambhir, Juan Leon, Ben Lopman

**Affiliations:** Rollins School of Public Health, Emory University, Atlanta, Georgia, USA (K. Simmons, J. Leon, B. Lopman);; Centers for Disease Control and Prevention, Atlanta (K. Simmons, B. Lopman);; Imperial College London, London, UK (M. Gambhir)

**Keywords:** Norovirus, modeling, mathematical model, immunity, incidence, vaccination, vaccine development, viruses, enteric infections, acute gastroenteritis

## Abstract

The duration of immunity to norovirus (NoV) gastroenteritis has been believed to be from 6 months to 2 years. However, several observations are inconsistent with this short period. To gain better estimates of the duration of immunity to NoV, we developed a mathematical model of community NoV transmission. The model was parameterized from the literature and also fit to age-specific incidence data from England and Wales by using maximum likelihood. We developed several scenarios to determine the effect of unknowns regarding transmission and immunity on estimates of the duration of immunity. In the various models, duration of immunity to NoV gastroenteritis was estimated at 4.1 (95% CI 3.2–5.1) to 8.7 (95% CI 6.8–11.3) years. Moreover, we calculated that children (<5 years) are much more infectious than older children and adults. If a vaccine can achieve protection for duration of natural immunity indicated by our results, its potential health and economic benefits could be substantial.

Noroviruses (NoVs) are the most common cause of acute gastroenteritis (AGE) in industrialized countries. In the United States, NoV causes an estimated 21 million cases of AGE (*1*), 1.7 million outpatient visits (*2*), 400,000 emergency care visits, 70,000 hospitalizations (*3*), and 800 deaths annually across all age groups (*4*). Although the highest rates of disease are in young children, infection and disease occur throughout life (*5*), despite an antibody seroprevalence >50%, and infection rates approach 100% in older adults (*6*,^7^).

Frequently cited estimates of the duration of immunity to NoV are based on human challenge studies conducted in the 1970s. In the first, Parrino et al. challenged volunteers with Norwalk virus (the prototype NoV strain) inoculum multiple times. Results suggested that the immunity to Norwalk AGE lasts from ≈2 months to 2 years (*8*). A subsequent study with a shorter challenge interval suggested that immunity to Norwalk virus lasts for at least 6 months (*9*). In addition, the collection of volunteer studies together demonstrate that antibodies against NoV may not confer protection and that protection from infection (serologic response or viral shedding) is harder to achieve than protection from disease (defined as AGE symptoms) (*10**–**14*). That said, most recent studies have reported some protection from illness and infection in association with antibodies that block binding of virus-like particles to histo-blood group antigen (HBGA) (*13*,*14*). Other studies have also associated genetic resistance to NoV infections with mutations in the 1,2-fucosyltransferase (*FUT2*) gene (or “secretor” gene) (*15*). Persons with a nonsecretor gene (*FUT2*−/−) represent as much as 20% of the European population. Challenge studies have also shown that recently infected volunteers are susceptible to heterologous strains sooner than to homotypic challenge, indicating limited cross-protection (*11*).

One of many concerns with all classic challenge studies is that the virus dose given to volunteers was several thousand–fold greater than the small amount of virus capable of causing human illness (estimated as 18–1,000 virus particles) (*16*). Thus, immunity to a lower challenge dose, similar to what might be encountered in the community, might be more robust and broadly protective than the protection against artificial doses encountered in these volunteer studies. Indeed, Teunis et al. have clearly demonstrated a dose-response relationship whereby persons challenged with a higher NoV dose have substantially greater illness risk (*16*).

Furthermore, in contrast with results of early challenge studies, several observations can be made that, when taken together, are inconsistent with a duration of immunity on the scale of months. First, the incidence of NoV in the general population has been estimated in several countries as ≈5% per year, with substantially higher rates in children (*5*). Second, Norwalk virus (GI.1) volunteer studies conducted over 3 decades, indicate that approximately one third of genetically susceptible persons (i.e., secretor-positive persons with a functional *FUT2* gene) are immune (Table 1) (*18**,**20**,**22*). The point prevalence of immunity in the population (i.e., population immunity) can be approximated by the incidence of infection (or exposure) multiplied by the duration of immunity. If duration of immunity is truly <1 year and incidence is 5%, <5% of the population should have acquired immunity at any given time. However, challenge studies show population immunity levels on the order of 30%–45%, suggesting that our understanding of the duration of immunity is incomplete (*8*,*11*,*17*,*18*). HBGA–mediated lack of susceptibility may play a key role, but given the high seroprevalence of NoV antibodies and broad diversity of human HBGAs and NoV, HBGA–mediated lack of susceptibility cannot solely explain the discrepancy between estimates of duration of immunity and observed NoV incidence. Moreover, population immunity levels may be driven through the acquisition of immunity of fully susceptible persons or through boosting of immunity among those previously exposed.

**Table 1:** Summary of literature review of Norwalk virus volunteer challenge studies*

Study	All				Secretor positive				Secretor negative		Strain
	No. challenged	No. (%) infected	No. (%) AGE		No. challenged	No. (%) infected	No. (%) AGE		No. challenged	No. (%) infected	
Dolin 1971 (*10*)	12		9 (75)								SM
Wyatt 1974 (*11*)†	23		16 (70)								NV, MC, HI
Parrino 1977 (*8*)†	12		6 (50)								NV
Johnson 1990 (*17*)†	42	31 (74)	25 (60)								NV
Graham 1994 (*12*)	50	41 (82)	34 (68)								NV
Lindesmith 2003 (*18*)	77	34 (44)	21 (27)		55	35 (64)	21 (38)		21	0	NV
Lindesmith 2005 (*19*)	15	9 (60)	7 (47)		12	8 (67)			3	1 (33)	SM
Atmar 2008 (*20*)	21	16 (76)	11 (52)		21	16 (76)	11 (52)				NV
Leon 2011 (*21*)‡	15	7 (47)	5 (33)		15	7 (47)	5 (33)				NV
Atmar 2011 (*14*)‡	41	34 (83)	29 (71)		41	34 (83)	29 (71)				NV
Seitz 2011 (*22*)	13	10 (77)	10 (77)		13	10 (77)	10 (77)			1 (5.6)	NV
Frenck 2012 (*23*)	40	17 (42)	12 (30)		23	16 (70)	12 (52.1)		17		GII.4

*AGE, acute gastroenteritis; SM, Snow Mountain virus; NV, Norwalk virus; MC, Montgomery County virus; HI, Hawaii virus; GII.4, genogroup 2 type 4. †Only includes initial challenge, not subsequent re-challenge. ‡Only includes placebo or control group.

In this study, we aimed to gain better estimates of the duration of immunity to NoV by developing a community-based transmission model that represents the transmission process and natural history of NoV, including the waning of immunity. The model distinguishes between persons susceptible to disease and those susceptible to infection but not disease. We fit the model to age-specific incidence data from a community cohort study. However, several factors related to NoV transmission remain unknown (e.g., the role asymptomatic persons who shed virus play in transmission). Therefore, we constructed and fit a series of 6 models to represent the variety of possible infection processes to gain a more robust estimate of the duration of immunity. This approach does not consider multiple strains or the emergence of new variants, so we are effectively estimating minimum duration of immunity in the absence of major strain changes. 

## Methods

### Model Design

**Figure 1:**
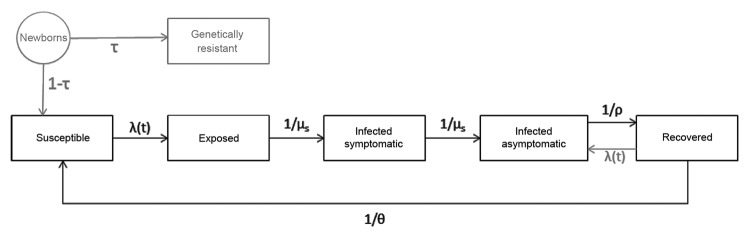
Model schematic illustrating the immunity and infection states of the population with respect to norovirus (NoV) infection and the flows between those states. Persons are born directly into the susceptible pool, become exposed at the force of infection, and then progress through symptomatic and asymptomatic stages before arriving in the recovered compartment, which represents immunity to disease, but not necessarily to infection. As such, from the recovered compartment, persons can become asymptomatically infected at the force of infection or can become susceptible to disease once again through the waning of immunity. For the sake of simplicity, deaths from all categories equal to the incoming births are not shown but are included in the model code. In 1 iteration of the model (scenario E), a compartment is included that represents a class of persons who are born with genetic resistance (in gray to represent absence in all other model iterations) to NoV infection.

We developed a deterministic dynamic transmission model with age structure that tracks the population with respect to NoV infection and immunity status (Figure 1; Technical Appendix). Here we describe the basic structure of the model (model A), which forms the basis for 5 other iterations (models B–F, described below). The models track 5 classes of persons: 1) susceptible to infection and disease (S), 2) exposed but not yet symptomatic (E), 3) infected with symptoms (I), 4) infected but asymptomatic (A), and 5) immune to disease, but not infection (R). In model D, we included an additional class for genetically resistant persons (G).

We assume that maternal immunity is negligible because the youngest age class includes children ages 0–4 years; as such, newborns in all models except model D enter directly into S class. In model D, genetically resistant persons bypass the S class and remain resistant for life, although they make contacts and are included in calculations of incidence for model-fitting purposes, because all persons (not just those susceptible) were included in the empirical studies to which the model was fit. All persons in the S class can be infected at rate λ(t) (the force of infection) and move into the E class. They then progress from the E class into the I class (symptomatic) at a rate intensely proportional to the incubation period (1/μ_s_)_._ We are thus assuming that when a susceptible (S) person becomes infected, disease will later develop and that all first infections are symptomatic. Persons then recover at a rate inversely proportional to duration of illness (1/μ_a_), at which point they are shedding asymptomatically (A). Infection then ends at a rate inversely proportional to duration of shedding (ρ), after which the person is assumed to have cleared the infection and is recovered from symptoms and that the person’s immune system protects from further disease (R).

Consistent with the understanding of NoV host response, in our model, NoV-specific immunity is not life-long and we allow 2 pathways out of R class. First, persons can become asymptomatically infected by cycling back into the A class at the same force of infection to which S persons are subjected [λ(t)]. As such, R class represents a type of immunity in which persons are subject to infection but not disease—they can become asymptomatically infected and shed virus in stool specimens, but symptoms of AGE do not develop. Persons in R class can also lose their immunity to disease through the waning process, whereby they become fully susceptible again at a rate of 1/θ. θ is a fitted parameter (described below). Births and deaths are assumed to be equal and occur at a constant rate throughout the year. Static model inputs are detailed in Table 2.

**Table 2:** Fixed input parameters for each model scenario for duration of immunity to norovirus gastroenteritis*

Parameter	Symbol	Model	Source

A	B	C	D	E	F
Life expectancy, y	NA	76	76	76	76	76	76	CDC FastStats (*24*)
Duration of incubation, d	μ_s_	1	1	1	1	1	1	Atmar et al., 2008 (*20*)
Duration of symptoms, d	μ_a_	2	2	2	2	2	2	Atmar et al., 2008 (*20*)
Duration of asymptomatic infection, d	ρ	10	10	10	10	10	10	Rockx et al., 2002 (*25*)
Relative infectiousness during incubation period	NA	0	0.05	0.25	0	0	0	Sukhrie et al., 2010 (*26*)
Relative infectiousness during asymptomatic infection period	NA	0	0.05	0.25	0	0	0	Sukhrie et al., 2010 (*26*)
Proportion of population genetically resistant	τ	0	0	0	0.2	0	0	Lindesmith et al., 2003 (*18*)
Strains included		All	All	All	All	GII.4 only	All	Rosenthal et al., 2011 (*27*)
Boosting of immunity by asymptomatic infection?	NA	Yes	Yes	Yes	Yes	Yes	No	

*NA, not applicable; CDC, Centers for Disease Control and Prevention. Model A, only symptomatic infectiousness; model B, presyymptomatic and postsymptomatic infectiousness (low); model C, presymptomatic and postsymptomatic infectiousness (high); model D, innate genetic resistance; model E, genogroup 2 type 4 (GII.4); model F, no immune boosting by asymptomatic infection.

In this baseline model (Figure 1), we assume that only symptomatic (I) persons contribute to transmission, so λ(t) is a function of the number of susceptible persons, the age-specific contact rate β_i,_ the prevalence of infection I(t), and the probability of transmission, given contact (Technical Appendix). We allow for children <5 years old to have a different, presumably higher, level of infectiousness (q_1_) than older children and adults (q_2_) (Table 3).

**Table 3:** Duration of immunity, fitted parameter estimates, and log-likelihood and basic reproductive number for models of duration of immunity to norovirus gastroenteritis

Parameter	Symbol	Model A	Model B	Model C	Model D	Model E	Model F
Duration of immunity, y	θ	5.1 (3.9–6.5)	5.1 (4.0– 6.7)	8.7 (6.8–11.3)	4.1 (3.2–5.1)	7.6 (5.6–8.0)	5.1 (3.9–6.6)
Probability of transmission per infected contact, 0–4 y	q_1_	0.25 (0.21–0.31)	0.18 (0.15–0.21)	0.37 (0.14–0.91)	0.35 (0.27–0.44)	0.23 (0.19–0.25)	0.25 (0.21–0.31)
Probability of transmission per infected contact, >5 y	q2	0.050 (0.042–0.055)	0.036 (0.032–0.039)	0.094 (0.078–0.114))	0.062 (0.057–0.066)	0.051 (0.47–0.056)	0.050 (0.046–0.054)
Negative log likelihood		615.497	613.905	663.052	616.597	611.509	615.375
Annual incidence, %†		5.2	5.3	5.5	5.1	3.8	5.2
Basic reproductive number (all ages)	R_0_	1.79	1.64	3.34	1.88	1.73	1.79
Basic reproductive number (0–4 y)	R_0_	4.33	3.98	6.41	4.84	3.98	4.33

*Model A, only symptomatic infectiousness; model B, presyymptomatic and postsymptomatic infectiousness (low); model C, presymptomatic and postsymptomatic infectiousness (high); model D, innate genetic resistance; model E, genogroup 2 type 4 (GII.4); model F, no immune boosting by asymptomatic infection. †Compared with an observed annual incidence 4.5% from Phillips et al. (*5*), except for model E, which should be compared with norovirus GII.4-specific incidence of 3.2%.

### Model Scenarios

Our first model incorporated several simplifications (e.g., that the entire population is genetically susceptible) for which considerable uncertainty exists (e.g., that immunity to 1 strain of NoV protects against other strains). Therefore, we set up several scenarios to explore the effects on duration of immunity estimates of pre- and postsymptomatic infectiousness, genetic resistance within a portion of the population, and whether immunity to NoV is strain specific (Table 2). 

#### Model A: Symptomatic Individuals Infectious

In model A, described in the previous section, only symptomatic individuals are infectious. This model provides the basis for the 5 following iterations. 

#### Model B: Presymptomatic and Postsymptomatic Infectiousness (Low)

Presymptomatic persons (E) have been observed to transmit NoV (*28*), although how often this occurs is not known. Also, exposed, but not-yet-symptomatic, persons (E) are 5% as infectious as symptomatic persons (*26*). Because they incubate the virus for only 1 day (1/2 as long as the symptomatic phase), they are 2.5% as infectious as a symptomatic case-patient over the course of their incubation period. Persons may shed virus after resolution of symptoms and may also become infected and shed virus without exhibiting symptoms. Again, their importance in transmission has not been quantified. Sukhrie et al. have demonstrated that asymptomatic shedders can transmit the virus, but they do so at lower levels than symptomatic persons (*26*,*29*). In this scenario, asymptomatic (A) and presymptomatic (E) persons are 5% as infectious as symptomatic persons. Because the mean duration of shedding is 10 days, asymptomatic and presymptomatic persons have a cumulative infectiousness of 25% compared with symptomatic persons (Table 2).

#### Model C: Presymptomatic and Postsymptomatic Infectiousness (High)

This model has the same structure as model B. However, persons in the exposed (E) and asymptomatic (A) compartments are 25% as infectious as symptomatic persons.

#### Model D: Innate Genetic Resistance

In model D, we assume that 20% of the population is completely resistant to infection and disease (i.e., they have the nonsecretor phenotype), and therefore play no role in the transmission process (Figure 1) (*18*). They do, however, continue to make contact with other persons and are included in empirical incidence estimates, so the whole population is included in this model, even though 20% cannot become infected. This model includes a separate class of persons born with complete genetic resistance (G).

#### Model E: Genogroup 2 Type 4 (GII.4)

In Models A–D, we assume that all NoVs are antigenetically indistinguishable, since the degree of strain specificity of NoV immunity is not well understood. Model E tests the sensitivity of that assumption by including only GII.4 infections, which have been the predominant circulating strain for the past decade. We multiplied incidence data by 0.72 (an estimate of the proportion of all NoV AGE caused by GII.4 viruses) (*32*) to represent only GII-4 cases and subsequently refitted the model. This model assumes that GII.4 viruses are antigenically distinct from non-GII.4 NoVs and that all GII.4 viruses are antigenically indistinguishable (*33*).

#### Model F: No Immune Boosting by Asymptomatic Infection

Persons do not move from the recovered (R) to asymptomatic (A) compartments. The only pathway out of the R class is through waning of immunity to become susceptible (S) again.

### Data and Model Fitting

We fit the model to age-specific incidence from the Study of Infectious Intestinal Disease in England (*5*) and the size of the adult (defined as 15– 44 years of age) population immune at endemic equilibrium by allowing the transmission probabilities (q_i_s) and duration of immunity (θ) to vary during the fitting process. Size of the immune population was estimated from a literature review of challenge studies (Table 1).

We calculated the log-likelihood of the data under each model by assuming Poisson distributions with mean equal to the number of model-predicted cases for symptomatic NoV incidence in each age group and number of immune persons in the adult age group (Technical Appendix). Both incidence and population immune were treated as count data, on the basis of the size of the study population in the study in England (*5*) and the cumulative number of subjects included in challenge studies. The best-fitting parameter set maximized the log-likelihood of the age-stratified time series for the given set of estimated and fixed parameters (*34*). We calculated 95% CIs for each parameter (in each model) and generated a likelihood profile by holding a given parameter constant at a series of values and refitting the model. The upper and lower values were found by using the likelihood ratio test to determine at which parameter value the model converged on a significantly worse fit.

Because seasonality is a defining characteristic of NoV infection, we added seasonal forcing variables to visually inspect whether US outbreak patterns as described by Yen et al. (*35*) could be captured. We allowed the transmission coefficient (β_1_) to vary by 6% over the course of the year. However, because including seasonality did not qualitatively change our estimate of the duration of immunity, we excluded it in favor of a more parsimonious model.

## Results

**Figure 2:**
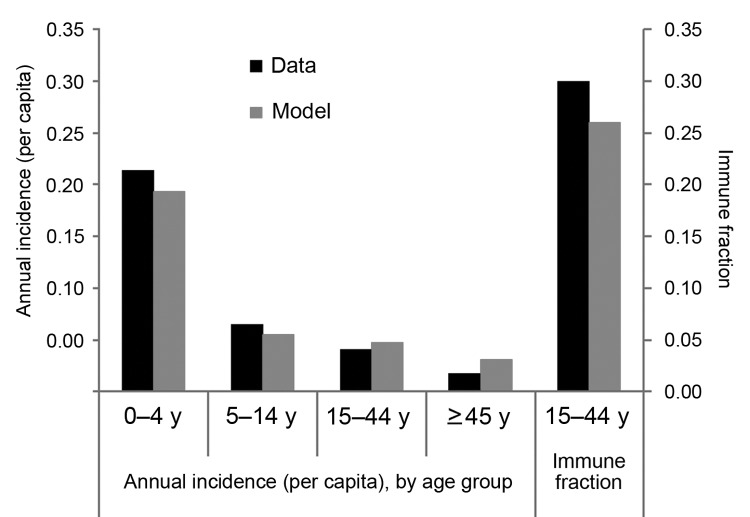
Age-specific annual incidence of norovirus gastroenteritis, observed (black) and model predicted (red). These results are for model B (which includes presymptomatic and postsymptomatic infectiousness).

All models provided a qualitatively good fit to the crude incidence data, ranging from 5.1% (models D and E) to 5.5% (model C) per year, compared with the observed 4.5% per year (Table 3; Figure 2; Technical Appendix Figure). All models also captured the decreasing incidence by age; model B was best able to represent the overall incidence and the high incidence in children <5 years of age (21.4% observed; 19.3% fitted), and model B roughly captured the incidence in the groups >45 years of age. Model C provided a worse fit than models A, B, D, or F. Model E could not be readily compared because it is fitted to a different incidence case count. Although model B was not a significantly better fit than A, D, or F, it did have the smallest negative log-likelihood, so we used model B for subsequent results, unless stated otherwise.

The R_0_ (basic reproductive number) for all models ranged from 1.64 to 1.88, except in model C, which had an R_0_ of 7.16. R_0_ for children 0–4 years of age was 15.22, substantially higher than for persons >5 years (R_0_ = 0.89) (model B, Table 3).

In model A, the duration of immunity to NoV was estimated at 5.1 years (95% CI 3.9–6.5; Table 3). The duration of immunity estimated in model B was essentially the same as in model A at 5.1 years (95% CI 4.0–7.6). When the infectiousness of asymptomatic persons was increased in model C, estimate of duration of immunity increased substantially, to 8.7 (95% CI 4.0–11.3). Duration of immunity estimated in model D, in which transmission was effectively restricted to 80% of the population, was 4.1 years (95% CI 3.2–5.1). In model E, which was essentially fitted to a lower incidence to reflect only GII.4 transmission, duration of immunity was estimated at 7.6 years (95% CI 5.6–8.0). Model F, which did not allow subclinical infection to boost immunity, resulted in a duration of immunity estimate of 5.1 years (95% CI 3.9–6.6). Note that the transmission parameters (q_i_s) fell into 3 relative patterns: lower (model B), middle (models A, E, and F), and high (models C and D). These differences in transmissibility partly explain why the duration of immunity estimates are not more divergent between models. 

**Figure 3:**
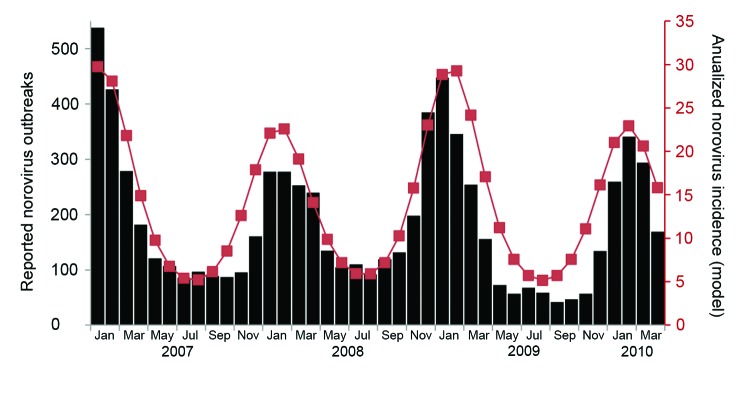
Norovirus gastroenteritis outbreak patterns from 30 US states, January 2007–April 2010 (white bars) and predicted annualized monthly incidence for all age groups (black line). These results are for model B (which includes presymptomatic and postsymptomatic infectiousness) and, for this illustration, seasonal forcing (*32*).

With mild seasonal forcing (6% seasonal variation in transmission probabilities), the model captures fluctuations in disease incidence similar to those reported from outbreaks in 30 US states during 2007–2010 (Figure 3). Seventy-three percent of cases were estimated to occur during October–March, compared with 73% observed in the United States during October–March from 2007 to 2010. 

## Discussion

The goal of this study was to gain a better estimate of the duration of immunity to NoV AGE, and our results suggest that it is longer than was previously understood. We modeled a range of possible infection and immunity processes to capture the unknown aspects of the transmission process, and from these models, we estimate a mean duration of immunity ranging from ≈4 to 8 years. Variations in duration of immunity between models can be traced to inclusion of presymptomatic and postsymptomatic states in the model scenarios. The best-fit models converge on a much higher infectiousness and R_0_ for young children (<5 years) than for older children and adults. This finding is consistent with observational studies that found contact with a symptomatic child to be the prime risk factor for NoV infection for children and adults (*5*,*30*) and suggests that young children have a key role in the transmission of NoV to all age groups. Children have relatively high rates of contact with both other children and adults, and because of their lower levels of hygiene, they are likely to be more infectious than adults, given contact (*31*).

In our study, the models produced strong quantitative fits to the empirical data on incidence and population immunity, as well as on seasonality. Results suggest that parameter estimates are not overly sensitive to structural uncertainties, such as the role of asymptomatic shedding in disease transmission, at least within the range of the fixed parameters we have considered. The possible exception is model C, which resulted in a much higher R_0_ than the other models and previous estimates, suggesting that asymptomatic persons are unlikely to be as infectious as they were parameterized to be in this scenario. Because the exact structure of immunity in the general population is unknown, our study sought to elucidate that structure rather than identify exact values for the various parameters included in each model scenario.

Several caveats should be borne in mind when interpreting these results. First, perhaps most critical, our model assumes that immunity is to disease (e.g., symptoms) rather than to infection. As such, so-called immune persons are still subject to becoming infected but they do not show symptoms. Infection without symptoms is a common outcome of exposure, as shown by volunteer studies and point prevalence of asymptomatic infection detected in the general population, which can be as high as 30% (*5**,**18**,**19*). In effect, our model allows for boosting of immunity by cycling between the recovered (R) and infectious asymptomatic (A) compartments. However, our estimates of duration of immunity pertain to time spent in the immune state from time of most recent symptomatic infection. If a person repeatedly became asymptomatically infected (moves from R to A class), that person would effectively be immune to disease for longer than a person without successive asymptomatic/subclinical infections. The duration of immunity estimates are therefore conservative with respect to total time a person is protected from disease.

Second, with this single-strain model, we assume that all NoVs are antigenically indistinguishable and that infection with 1 NoV provides protection against all others. This is not strictly true (*11*), but data are not available on cross-protection to a range of NoV strains circulating at a particular time. As an extreme simplification of this process, we modeled GII.4 viruses on the assumption that they comprise 72% of observed incidence and are an antigenically homotypic genotype, essentially acting as a separate virus. However, GII.4 viruses are antigenically distinct from other GII viruses, and every few years, new GII.4 strains emerge that escape acquired population immunity. Over the past 15 years, at least 2 immune escape variants of GII.4 have emerged (in 2002 and 2006) (*33*). Although our estimate of duration of immunity (>4 years) may be compromised by this assumption, that novel GII.4s emerge once every 4 years or so would still suggest a role for the duration of immunity on the scale of years. Immunity gained through exposure to the prevalent strain would persist past emergence of a new strain, even though such protection could be effectively useless against the new strain.

These findings could ultimately have implications for vaccine policy. Empirical studies strongly document that children have the highest incidence of disease. Our results suggest that young children play a dominant role in the transmission process. Therefore, vaccinating young children is likely to result in both the greatest direct and indirect benefits. This conclusion is at odds with the current direction of vaccine development, which is increasingly focused on demonstrating safety and efficacy in older age groups (*36*,*37*). Future modeling studies could explicitly examine the potential direct and indirect benefits of vaccinating different age groups. Moreover, severe disease disproportionately occurs among the elderly (despite their lower incidence of disease), but the elderly are difficult to successfully immunize for both programmatic and immunologic reasons. Therefore, future modeling studies should address the question of whether severe disease outcomes could best be prevented directly, by vaccinating the elderly, or indirectly, by vaccinating children (*38**–**40*). Our study provides estimates of the infectiousness of children <5 years of age and adults (with the former being much more infectious) on which to base such simulations.

Because these results suggest a longer duration of protection than previously estimated, they support the continued development of NoV vaccines. A short duration of protection (<1 year, for example) would be a major impediment for widespread use of a NoV vaccine because it would have to be given frequently, and the distribution would be expensive and logistically difficult (e.g., willingness for annual vaccination). However, if duration of immunity and possibly vaccine protection are indeed on the order of 5 years, as this study suggests, the cost-benefits and health gains per person vaccinated could be substantially greater than previously estimated (*41*).

Our findings represent a substantial departure from current estimates of the duration of immunity to NoV. As noted, our models make several potentially influential simplifying assumptions. However, these models, grounded in observational evidence on age-specific incidence, seasonality of disease, and levels of population immunity, may be more realistic than results of re-challenge studies, which have formed the basis of current estimates. Specifically, this analysis suggests that the large dose or type (GI.1) delivered to volunteers in the classic challenge studies was unrepresentative of natural exposure to common contemporary strains. Because a robust duration of protection is likely crucial for the success of vaccines, future trials could consider following-up at least a subset of participants for several years either for natural disease or by challenge, providing an empirical test of these modeling results.

Technical Appendix Description of differential equations used in a deterministic dynamic transmission model with age structure that tracks the population with respect to norovirus infection and immunity status.

## References

[1] Scallan E, Griffin PM, Angulo FJ, Tauxe RV, Hoekstra RM. Foodborne illness acquired in the United States—unspecified agents. Emerg Infect Dis. 2011;17:16–22. 10.3201/eid1701.P21101 21192849 PMC3204615

[2] Hall AJ, Rosenthal M, Gregoricus N, Greene SA, Ferguson J, Henao OL, Incidence of acute gastroenteritis and role of norovirus, Georgia, USA, 2004–2005. Emerg Infect Dis. 2011;17:1381–8 . 21801613 10.3201/eid1708.101533 PMC3381564

[3] Lopman B, Gastañaduy P, Park GW, Hall AJ, Parashar UD, Vinjé J. Environmental transmission of norovirus gastroenteritis. Curr Opin Virol. 2012;2:96–102. 10.1016/j.coviro.2011.11.005 22440972

[4] Hall AJ, Curns AT, McDonald LC, Parashar UD, Lopman BA. The roles of *Clostridium difficile* and norovirus among gastroenteritis-associated deaths in the United States, 1999–2007. Clin Infect Dis. 2012;55:216–23. 10.1093/cid/cis386 22491338

[5] Phillips G, Tam CC, Conti S, Rodrigues LC, Brown D, Iturriza-Gomara M, Community incidence of norovirus-associated infectious intestinal disease in England: improved estimates using viral load for norovirus diagnosis. Am J Epidemiol. 2010;171:1014–22. 10.1093/aje/kwq021 20360244

[6] Parker SP, Cubitt WD, Jiang XJ, Estes MK. Seroprevalence studies using a recombinant Norwalk virus protein enzyme immunoassay. J Med Virol. 1994;42:146–50 . 10.1002/jmv.1890420209 8158109

[7] Jing Y, Qian Y, Huo Y, Wang LP, Jiang X. Seroprevalence against Norwalk-like human caliciviruses in Beijing, China. J Med Virol. 2000;60:97–101. 10.1002/(SICI)1096-9071(200001)60:1<97::AID-JMV16>3.0.CO;2-D 10568770

[8] Parrino TA, Schreiber D, Trier J, Kapikian A, Blacklow N. Clinical immunity in acute gastroenteritis caused by Norwalk agent. N Engl J Med. 1977;297:86–9. 10.1056/NEJM197707142970204 405590

[9] Johnson PC, Mathewson JJ, DuPont HL, Greenberg HB. Multiple-challenge study of host susceptibility to Norwalk gastroenteritis in US adults. J Infect Dis. 1990;161:18–21, ONUDL 2153184. 10.1093/infdis/123.3.307 2153184

[10] Dolin R, Blacklow NR, DuPont H, Formal S, Buscho RF, Kasel J. Transmission of acute infectious nonbacterial gastroenteritis to volunteers by oral administration of stool filtrates. J Infect Dis. 1971;123:307–12. 10.1093/infdis/123.3.307 5111887

[11] Wyatt RG, Dolin R, Blacklow NR, DuPont HL, Buscho RF, Thornhill TS, Comparison of three agents of acute infectious nonbacterial gastroenteritis by cross-challenge in volunteers. J Infect Dis. 1974;129:709–14. 10.1093/infdis/129.6.709 4209723

[12] Graham DY, Jiang X, Tanaka T, Opekun AR, Madore HP, Estes MK. Norwalk virus infection of volunteers: new insights based on improved assays. J Infect Dis. 1994;170:34–43. 10.1093/infdis/170.1.34 8014518

[13] Reeck A, Kavanagh O, Estes MK, Opekun AR, Gilger MA, Graham DY, Serological correlate of protection against norovirus-induced gastroenteritis. J Infect Dis. 2010;202:1212–8. 10.1086/656364 20815703 PMC2945238

[14] Atmar RL, Bernstein DI, Harro CD, Al-Ibrahim MS, Chen WH, Ferreira J, Norovirus vaccine against experimental human Norwalk virus illness. N Engl J Med. 2011;365:2178–87 . 10.1056/NEJMoa1101245 22150036 PMC3761795

[15] Le Pendu J, Ruvo N, Kindberg E, Svensson L. Mendelian resistance to human norovirus infections. Semin Immunol. 2006;18:375–86. 10.1016/j.smim.2006.07.009 16973373 PMC7129677

[16] Teunis PFM, Moe CL, Liu P, Miller SE, Lindesmith L, Baric RS, Norwalk virus: how infectious is it? J Med Virol. 2008;80:1468–76. 10.1002/jmv.21237 18551613

[17] Johnson PC, Mathewson JJ, Dupont HL, Greenberg HB. Study of host susceptibility to Norwalk gastroenteritis in US adults. J Infect Dis. 1990;161:18–21. 10.1093/infdis/161.1.18 2153184

[18] Lindesmith L, Moe C, Marionneau S, Ruvoen N, Jiang X, Lindblad L, Human susceptibility and resistance to Norwalk virus infection. Nat Med. 2003;9:548–53. 10.1038/nm860 12692541

[19] Lindesmith L, Moe C, Lependu J, Frelinger JA, Treanor J, Baric RS. Cellular and humoral immunity following Snow Mountain virus challenge. J Virol. 2005;79:2900–9. 10.1128/JVI.79.5.2900-2909.2005 15709009 PMC548455

[20] Atmar RL, Opekun AR. Gilger MA, Estes MK, Crawford SE, Neill FH, et al. Norwalk virus shedding after experimental human infection. Emerg Infect Dis. 2008;14:1553–7. 10.3201/eid1410.080117 PMC2609865 18826818

[21] Leon JS, Kingsley DH, Montes JS, Richards GP, Lyon GM, Abdulhafid GM, Randomized, double-blinded clinical trial for human norovirus inactivation in oysters by high hydrostatic pressure processing. Appl Environ Microbiol. 2011;77:5476–82. 10.1128/AEM.02801-10 21705552 PMC3147477

[22] Seitz SR, Leon JS, Schwab KJ, Lyon GM, Dowd M, McDaniels M, Norovirus infectivity in humans and persistence in water. Appl Environ Microbiol. 2011;77:6884–8. 10.1128/AEM.05806-11 21856841 PMC3187119

[23] Frenck R, Bernstein DI, Xia M, Huang P, Zhong W, Parker S, Predicting susceptibility to norovirus GII. 4 by use of a challenge model involving humans. J Infect Dis. 2012;206:1386–93. 10.1093/infdis/jis514 22927452

[24] Centers for Disease Control and Prevention. FastStats–Life expectancy [cited 2012 Sep 13]. http://www.cdc.gov/nchs/fastats/lifexpec.htm

[25] Rockx B, De Wit M, Vennema H, Vinjé J, De Bruin E, Van Duynhoven Y, Natural history of human calicivirus infection: a prospective cohort study. Clin Infect Dis. 2002;35:246–53. 10.1086/341408 12115089

[26] Sukhrie FHA, Siebenga JJ, Beersma MFC, Koopmans M. Chronic shedders as reservoir for nosocomial transmission of norovirus. J Clin Microbiol. 2010;48:4303–5. 10.1128/JCM.01308-10 20810762 PMC3020829

[27] Rosenthal NA, Lee LE, Vermeulen BAJ, Hedberg K, Keene WE, Widdowson M-A, Epidemiological and genetic characteristics of norovirus outbreaks in long-term care facilities, 2003–2006. Epidemiol Infect. 2011;139:286–94. 10.1017/S095026881000083X 20412611

[28] Ozawa K, Oka T, Takeda N, Hansman GS. Norovirus infections in symptomatic and asymptomatic food-handlers in Japan. J Clin Microbiol. 2007;45:3996–4005. 10.1128/JCM.01516-07 17928420 PMC2168587

[29] Sukhrie FHA, Teunis P, Vennema H, Copra C, Beersma MFCT, Bogerman J, Nosocomial transmission of norovirus is mainly caused by symptomatic cases. Clin Infect Dis. 2012;54:931–7. 10.1093/cid/cir971 22291099

[30] de Wit MAS, Koopmans MP, Kortbeek LM, Wannet WJ, Vinjé J, Van Leusden F, Sensor, a population-based cohort study on gastroenteritis in the Netherlands: incidence and etiology. Am J Epidemiol. 2001;154:666–74. 10.1093/aje/154.7.666 11581101

[31] Mossong J, Hens N, Jit M, Beutels P, Auranen K, Mikolajczyk R, Social contacts and mixing patterns relevant to the spread of infectious diseases [cited 2012 Jul 13]. PLoS Med. 2008;5:e74. 10.1371/journal.pmed.0050074 18366252 PMC2270306

[32] Vega E, Barclay L, Gregoricus N, Williams K, Lee D, Novel surveillance network for norovirus gastroenteritis. Emerg Infect Dis. 2011;17:1389–95 . 21801614 10.3201/eid1708.101837 PMC3381557

[33] Siebenga JJ, Vennema H, Zheng D-P, Vinjé J, Lee BE, Pang X-L, Norovirus illness is a global problem: emergence and spread of norovirus GII.4 variants, 2001–2007. J Infect Dis. 2009;200:802–12. 10.1086/605127 19627248

[34] White LJ, Mandl JN, Gomes MGM, Bodley-Tickell AT, Cane PA, Perez-Brena P, Understanding the transmission dynamics of respiratory syncytial virus using multiple time series and nested models. Math Biosci. 2007;209:222–39. 10.1016/j.mbs.2006.08.018 17335858 PMC3724053

[35] Yen C, Wikswo ME, Lopman BA, Vinje J, Parashar UD, Hall AJ. Impact of an emergent norovirus variant in 2009 on norovirus outbreak activity in the United States. Clin Infect Dis. 2011;53:568–71. 10.1093/cid/cir478 21832262

[36] Thomas-Crusells J, McElhaney JE, Aguado MT. Report of the ad-hoc consultation on aging and immunization for a future WHO research agenda on life-course immunization. Vaccine. 2012;30:6007–12. 10.1016/j.vaccine.2012.07.025 22835737

[37] National Institutes of Health. ClinicalTrials.gov. Bivalent norovirus vaccine study [cited 2012 Sep 11]. http://clinicaltrials.gov/ct2/show/NCT01168401?term=norovirus+ligocyte&rank=2

[38] Zaman K, Roy E, Arifeen SE, Rahman M, Raqib R, Wilson E, Effectiveness of maternal influenza immunization in mothers and infants. N Engl J Med. 2008;359:1555–64. 10.1056/NEJMoa0708630 18799552

[39] Tamma PD, Ault KA, Rio C, Steinhoff MC, Halsey NA, Omer SB. Reviews Safety of influenza vaccination during pregnancy. Am J Obstet Gynecol. 2009;201:547–52. 10.1016/j.ajog.2009.09.034 19850275

[40] Steinhoff MC, Omer SB. Influenza immunization in pregnancy : antibody responses in mothers and infants. N Engl J Med. 2010;362:1644–6 . 10.1056/NEJMc0912599 20427817

[41] Bartsch SM, Lopman BA, Hall AJ, Parashar UD, Lee BY. The potential economic value of a human norovirus vaccine for the United States. Vaccine. 2012;30:7097–104. 10.1016/j.vaccine.2012.09.040 23026689 PMC3517973

