# Supplementary material for: Duration of Immunity to Norovirus Gastroenteritis
Source: Emerg Infect Dis. 2013 Aug;19(8):1260–7. doi: 10.3201/eid1908.130472 (PMC3739512; doi:10.3201/eid1908.130472)
Supplement: Technical Appendix — Description of differential equations used in a deterministic dynamic transmission model with age structure that tracks the population with respect to norovirus infection and immunity status. [file 13-0472-Techapp-s1.pdf]

Article DOI: http://dx.doi.org/10.3201/eid1908.130472

Duration of Immunity to Norovirus Gastroenteritis

Technical Appendix

This Technical Appendix describes the differential equations used in a deterministic dynamic transmission model with age structure that tracks the population with respect to norovirus infection and immunity status.

Model Equations

Our model is a system of ordinary differential equations, described as follows:

$$\frac{dGi}{dt} = B\tau - D\tau$$

τ is 0 is all models except E, so there is no genetically resist class in all other models.

$$\frac{dS_i}{dt} = B(1-\tau) + 1/\theta R_i - (\lambda i(t) + D)Si$$

$$\frac{dE_i}{dt} = \lambda(t)S_i - (1/\mu_s + D)Ei$$

$$\frac{dIS_i}{dt} = 1/\mu_s E_i - (1/\mu_a a + D)ISi$$

$$\frac{dIA_i}{dt} = 1/\mu_a IS_i + \lambda i(t)R_i - (1/\rho + D)IAi$$

The $\lambda i(t)R_i$ term is not included in model F

$$\frac{dR_i}{dt} = 1/\rho IA_i - (\lambda i(t) + 1/\theta + D)R_i$$

The $\lambda i(t)R_i$ term is not included in model F

where:

i = age group, defined as 0-4 years, 5-14 years, 15-45 years and 45+ years

G_i = individuals genetically resistant to NoV

S_i = individuals susceptible to NoV

E_i = individuals exposed to NoV

S_i = individuals infected with NoV and showing symptoms

A_i = individuals infected with NoV and not showing symptoms, but shedding virus in stool

R_i = individuals who have recovered from NoV and are no longer shedding virus

τ = proportion of individuals who are genetically resistant to NoV

B = number of births entering the system

D = number of deaths exiting the system

θ = the rate at which recovered individuals become susceptible again; the duration of immunity

$\lambda(t)$ = the force of infection; the rate at which susceptible or recovered individuals become infected

μ_s = the rate at which exposed individuals become infected symptomatic

μ_a = the rate at which infected symptomatic individuals become infected asymptomatic

ρ = the rate at which infected asymptomatic individuals recover

Seasonality

Norovirus has such a seasonal pattern that it was initially described as 'the winter vomiting disease.' Incidence peaks in the late winter and early spring, and most outbreaks are seen during this time. We accounted seasonal variation in transmissibility in the model as follows:

$$\beta(t)= \beta_0\ ((1+ \beta_1 \times \cos(2\pi t + \omega))$$

where β_0 is the mean of the transmission parameter, β_1 is the amplitude of its seasonal fluctuation and is the phase angle in years (t). The mean transmission parameter (β_1) depends on age-specific mixing and contact patterns of the population.

Force of infection (per Susceptible)

Since we had several scenarios with varying compartments for infected individuals, the expression for force of infection varied by scenario. These expressions are described below:

Scenarios A, D, E & F:

$$\lambda_i(\mathrm{t}) = \sum_{j=1}^{4} \mathrm{q}_i \mathrm{c}_{ij}(\mathrm{t})\mathrm{I}_i = \sum_{j=1}^{4} \beta_{ij}(\mathrm{t})\mathrm{I}_i$$

Scenario B:

$$\lambda_i(\mathrm{t}) = \sum_{j=1}^{4} \mathrm{q}_i \mathrm{c}_{ij}(\mathrm{t})(0.05\mathrm{E}_i + \mathrm{I}_i + 0.05\mathrm{A}_i) = \sum_{j=1}^{4} \beta_{ij}(\mathrm{t})(0.05\mathrm{E}_i + \mathrm{I}_i + 0.05\mathrm{A}_i)$$

Scenario C:

$$\lambda_i(\mathrm{t}) = \sum_{j=1}^{4} \mathrm{q}_i \mathrm{c}_{ij}(\mathrm{t})(0.25\mathrm{E}_i + \mathrm{I}_i + 0.25\mathrm{A}_i) = \sum_{j=1}^{4} \beta_{ij}(\mathrm{t})(0.25\mathrm{E}_i + \mathrm{I}_i + 0.25\mathrm{A}_i)$$

where i represents the various age groups and 0.05 or 0.025 is the value used to account for the differences in infectiousness between symptomatic individuals and those in the exposed and asymptomatic compartments. c_{ij} represents the contact rates between age group i and j, such that $\beta_{ij}(\mathrm{t}) = \mathrm{q}_i \mathrm{c}_{ij}(\mathrm{t})$ and is the rate at which two individuals come into effective contact with each other per unit time. All other terms are defined elsewhere in the manuscript or appendix.

Contact Rates

Contact rates were obtained from the European POLYMOD study (1) and adjusted to account for the age ranges in our model. We specifically used social contact rates from Great Britain. The rates used in the model are detailed in the Table below.

Technical Appendix Table: Contact rates used in the model, calculated from Mossong et al, 2007. Numbers represent the daily probability of someone in age group *i* coming in contact with someone in age group *j*. Row and column labels refer to the age groups used in the model.

	0-4 years	5-14 years	15-44 years	45+ years
**0-4 years**	7.13621e-7			
**5-14 years**	1.81279e-7	1.1697e-6		
**15-44 years**	1.67541e-7	2.2533e-7	3.51465e-7	
**45+ years**	7.03979e-8	9.5557e-8	1.65856e-7	2.03e-7

Fitting

The model fit was assessed by calculating the log-likelihood of the data under the assumption that the observations (monthly incidence in each age class ($i=1..4$) and the proportion immune ($i = 5$) at time t ($d_{i,t}$)) follow a Poisson distribution with mean $d_{i,t}$ as follows:

$$LL_{i,t} = -D_{i,t} + d_{i,t}\ln(D_{i,t}) - \sum_{j=1}^{d_{i,t}} \ln j$$

$$LL = \sum_{t}\sum_{i=1}^{5} LL_{i,t}$$

The best-fitting model was the one for which the set of estimated parameters { $\theta,q1,q2$} yielded the maximum log-likelihood. We fit the model after an initial burn-in period of 50 years to allow the model to reach equilibrium.

Calculation of R_0

We follow the approach outlined in the paper by Diekmann et al [1] to calculate the basic reproduction number for each of the models detailed in this study. This method requires us to construct a Next Generation Matrix (NGM) for our model system (outlined in the 'Model Equations' section of the Appendix) and calculate the eigenvalues of this matrix. R0 is then equal to the largest eigenvalue, or spectral radius, of the matrix. We go about constructing the NGM by first reducing the system to the infected states (i.e. the compartments labeled E, I, and A) so that we have a system with 12 state variables consisting of three infected states for each of the four age-groups. We then populate a Transmission matrix (T)—which includes all epidemiological ‘births’—and a Transition matrix (E)—which contains all flows between infected compartments.

If we first define a 'force of infection' matrix F as:

$$F = \begin{bmatrix} q_1.\beta_{11} & q_2.\beta_{12} & q_2.\beta_{13} & q_2.\beta_{14} \\ q_1.\beta_{21} & q_2.\beta_{22} & q_2.\beta_{23} & q_2.\beta_{24} \\ q_1.\beta_{31} & q_2.\beta_{32} & q_2.\beta_{33} & q_2.\beta_{34} \\ q_1.\beta_{41} & q_2.\beta_{42} & q_2.\beta_{43} & q_2.\beta_{44} \end{bmatrix}$$

Where the values β_{ij} are the elements of the age-dependent mixing matrix and the values q_1 and q_2 are the infectivity of those under and over 5 years, respectively, then the Transmission matrix T can be written as:

$$T = \begin{bmatrix} \mathrm{inf}_1.F & \mathrm{inf}_2.F & \mathrm{inf}_3.F \\ 0 & 0 & 0 \\ 0 & 0 & 0 \end{bmatrix}$$

where 0 indicates a 4 by 4 matrix of zeros, and inf1, inf2 and inf3 are the infectivity levels of each of the individuals in each of the infected compartments E, I, and A respectively.

And if we define:

$$\mu_S = \begin{bmatrix} \mu_S & 0 & 0 & 0 \\ 0 & \mu_S & 0 & 0 \\ 0 & 0 & \mu_S & 0 \\ 0 & 0 & 0 & \mu_S \end{bmatrix}, \mu_A = \begin{bmatrix} \mu_A & 0 & 0 & 0 \\ 0 & \mu_A & 0 & 0 \\ 0 & 0 & \mu_A & 0 \\ 0 & 0 & 0 & \mu_A \end{bmatrix} \text{ and } \rho = \begin{bmatrix} \rho & 0 & 0 & 0 \\ 0 & \rho & 0 & 0 \\ 0 & 0 & \rho & 0 \\ 0 & 0 & 0 & \rho \end{bmatrix}$$

Our Transition matrix E is given by:

$$E = \begin{bmatrix} -\mu_S & 0 & 0 \\ \mu_S & -\mu_A & 0 \\ 0 & \mu_A & -\rho \end{bmatrix}$$

The NGM K, is formulated as:

$$K = -T.E^{-1}$$

and the basic reproduction number is calculated as the largest eigenvalue of K .

Model fits to all scenarios

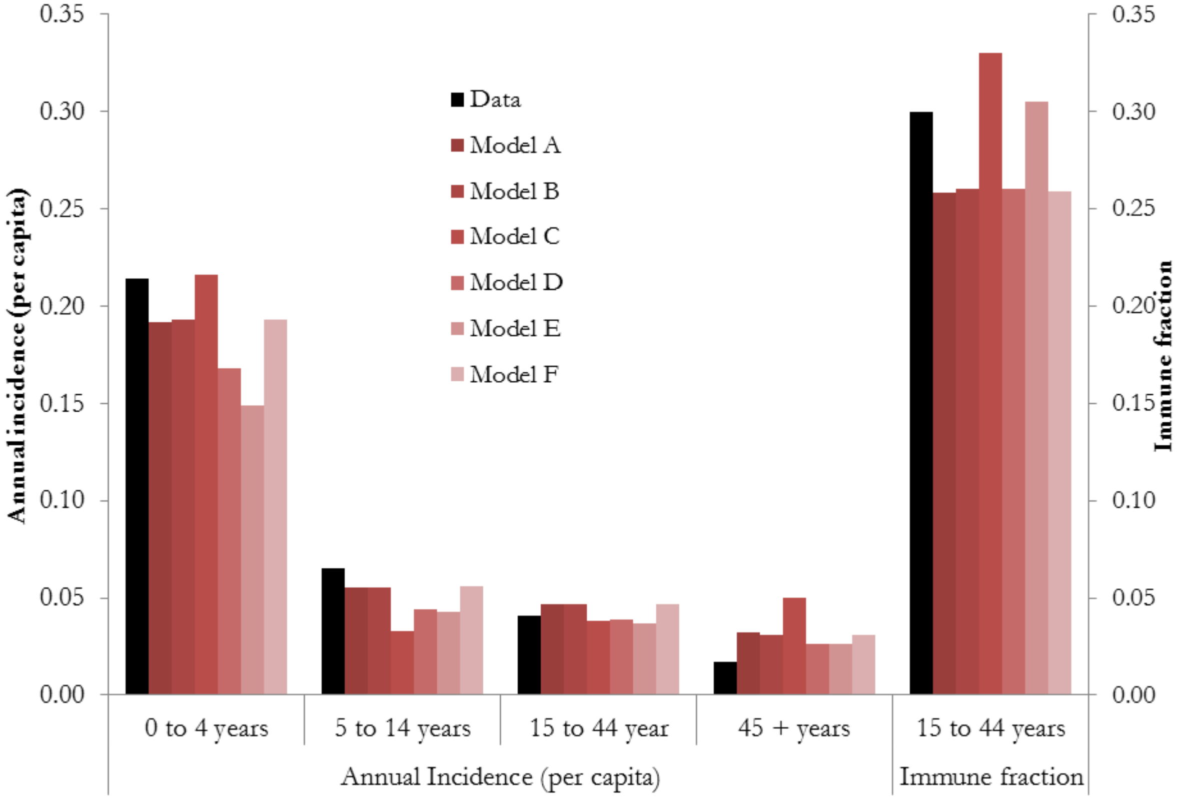

Technical Appendix Figure. Observed annual incidence (black) compared with output from the model (red bars) by age group, displayed by scenario.

Reference

1. Mossong J, Hens N, Jit M, Beutels P, Auranen K, Mikolajczyk R, et al. Social contacts and mixing patterns relevant to the spread of infectious diseases. PLoS medicine [Internet]. 2008 Mar 25 [cited 2012 Jul 13];5(3):e74. http://www.pubmedcentral.nih.gov/articlerender.fcgi?artid=2270306&tool=pmcentrez&rendertype=abstract
